# Electrospun Maltodextrin Fibers for Efficient Removal of Nanoparticles, Atenolol, and Crystal Violet: Preparation and Characterization

**DOI:** 10.1021/acsomega.5c03266

**Published:** 2025-10-15

**Authors:** Eya Ben Khalifa, Claudio Cecone, Boutheina Rzig, Giulia Mori, Federico Cesano, Mery Malandrino, Pierangiola Bracco, Giuliana Magnacca

**Affiliations:** † Department of Chemistry and NIS Interdepartmental Centre, 9314 University of Turin, Via Pietro Giuria 7, Torino 10125, Italy; ‡ Ecochimie Laboratory, National Institute of Applied Sciences and Technology (INSAT), 61796 University of Carthage, Tunis 1080, Tunisia

## Abstract

Maltodextrins are promising, sustainable, and low-cost materials suitable for environmental remediation applications. This study developed eco-friendly electrospun cross-linked fibers from maltodextrins (GLU2), combined with betaine, lysine, and cysteine. The resulting fibers exhibited a well-defined bead-free structure, good thermal stability, and successfully incorporated nitrogen functional groups. They were subsequently tested for removing water pollutants, including metal nanoparticles, crystal violet, and atenolol. Adsorption tests revealed that GLU2 fibers achieved 82% atenolol removal, although the performance was limited for silver nanoparticles, likely due to particle aggregation. The combination with lysine proved to be the most effective for gold nanoparticle removal, while cysteine-modified GLU2 fibers achieved up to 98% removal of crystal violet. These findings highlight the potential of amino acid-modified maltodextrin electrospun mats as effective adsorbents for water remediation.

## Graphical abstract

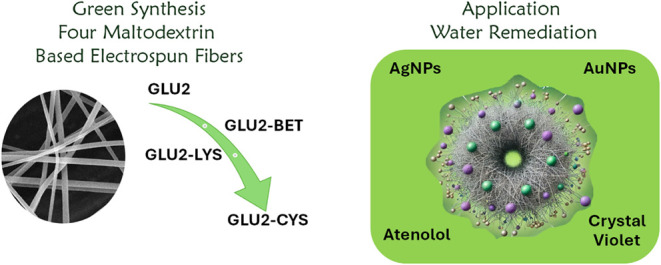

## 1 Introduction

The increasing concern over the adverse impact of anthropogenic contamination on water resources has driven efforts to develop effective control measures.^1,2^ Rapid industrialization has led to the release of diverse contaminants such as metal nanoparticles, pharmaceuticals, and dyes into aquatic environments.^3–5^ Silver nanoparticles are widely known for their antimicrobial properties and are used in applications including wound dressings, textiles, and disinfectants.^6–10^ Similarly, gold nanoparticles are applied in therapeutic fields, sensing, imaging, and wastewater treatment.^11,12^ Although metallic nanoparticles have promising applications, growing concerns about their potential toxicity, including cytotoxicity, oxidative stress, and inflammation, are emerging.^13–16^ Atenolol is among the most widely prescribed beta-blockers for cardiovascular diseases.^17^ The chemical resistance and high-water solubility of this drug contribute to its persistence in the environment, raising ecological concerns.^18^ Crystal violet is a cationic dye, widely used in the medical field as a biological stain, colorant, bacteriostatic, and antimicrobial agent.^19^ Exposure to water contaminated with this dye can result in serious health issues, including damage to the kidneys, liver, eyes, and skin irritation.^20^ Researchers have explored a range of materials for the abatement of these pollutants, such as activated carbon,^21–23^ activated sludge,^24^ clay,^25,26^ and graphene oxide.^27^ However, many of these conventional adsorbents are derived from fossil resources and present sustainability issues due to their long degradation times, often exceeding one hundred years. These limitations highlight the critical need to transition toward biodegradable and renewable materials that minimize ecological impact.^28,29^ Biobased materials like polysaccharides have gained growing attention in science and industry for their use in packaging, the food industry, biocomposites production, pharmaceuticals, medical applications, and environmental remediation.^30–34^ Maltodextrins, derived from starch via enzymatic conversion, are water-soluble, d-glucose-based polymers. Their cost-effectiveness and chemical versatility make them attractive building blocks for synthesizing cross-linked polymers.^35–42^ Additionally, they have proven successful in producing polysaccharide-based fibers through electrospinning.^43^ Electrospinning, an inexpensive and versatile processing technique, involves the production of fibers exploiting jets of polymer solutions obtained under high-voltage electric fields.^44^ Notably, the technique’s strength lies in the high surface-to-volume ratio of the produced fibrous mats. This technique is characterized by fiber diameters ranging from micrometers to the nanoscale.^45–47^ However, toxic or flammable organic solvents are commonly employed to dissolve the polymer, posing limitations to large-scale production due to safety and environmental concerns.^48–53^ Consequently, there is an active research focus on reducing or substituting hazardous substances, aligning with the principles of Green Chemistry.^54,55^ Stijnman et al.^56^ and Vargas-Campos et al.^57^ reported the use of water and ethanol/water mixtures as solvent media for the electrospinning of maltodextrins. Furthermore, the cross-linking of electrospun maltodextrin fibers containing proteins has been achieved by exploiting Maillard reactions.^58–62^ Functionalization with amino acids has become a suitable source for designing functional polymers. Their wide availability and high biocompatibility make them attractive for various applications. Previous studies have explored amino-acid-functionalized polysaccharides for biomedical applications. Ruggeri et al.^63^ developed maltodextrin-based fibers functionalized with polylysine and arginine for wound healing. Betaine has been grafted onto cotton fibers to study antibacterial and antiprotein adsorption properties. Dacrory et al.^64^ functionalized oxidized cellulose with glycine to investigate antifungal activity, while Hasanin et al.^65^ prepared a bioactive composite using L-phenylalanine and L-tryptophan grafted onto oxidized cellulose to evaluate antibacterial performance. However, to the best of our knowledge, the combination of lysine, cysteine, and betaine with maltodextrin-based electrospun fibers for environmental remediation, specifically for the removal of diverse contaminants, has not been previously reported. Our research group has recently conducted environmental studies on electrospun fibers based on dextrins, focusing on removing emerging contaminants from water.^66^ Furthermore, noteworthy research by Nthunya et al.^67^ and Huang et al.^68^ has demonstrated the efficacy of chitosan-based fibers in eliminating phenols and metal ions, respectively. Tran et al.^69^ have studied the efficiency of functionalized pectin fibers in mercury ion removal. Most of these studies focus on individual pollutant types, whereas comparative studies across different functionalized materials and multiple pollutant classes are limited. In this study, we address this gap by evaluating and comparing the adsorption performance of lysine, cysteine, and betaine functionalized maltodextrin fibers for the removal of three different classes of emerging water contaminants: metallic nanoparticles, pharmaceuticals (atenolol), and dyes (crystal violet).

With the above in mind, this study reports the preparation of four electrospun cross-linked fibers consisting of (i) plain maltodextrins, (ii) betaine-functionalized maltodextrins, (iii) L-cysteine-functionalized maltodextrins, and (iv) lysine-functionalized maltodextrins. The cross-linking of the electrospun mats, as well as the amino acid and betaine functionalization, was achieved by exploiting a one-step, environmentally friendly thermal treatment reaction involving citric acid and Maillard reactions. The incorporation of betaine, lysine, and cysteine into a maltodextrin mixture was aimed at preparing electrospun fibers with the appropriate functional groups necessary for effectively removing water pollutants. The resulting materials were thoroughly characterized and compared to evaluate how each amino acid affects the fiber properties. Furthermore, their adsorption performance toward metallic nanoparticles, atenolol, and crystal violet removal was studied and discussed.

## 2 Materials and Methods

### 2.1 Materials

Glucidex 2 (GLU2) was provided by Roquette Frères. GLU2 is a maltodextrin derived from the partial hydrolysis of corn starch. It possesses a dextrose equivalent of 2, a degree of branching (ratio of α-(1–6) linkages to the sum of α-(1–6) and α-(1–4) linkages) of 4.7%,^70^ and polydispersed molecular weights ranging from those of monomers and dimers up to approximately 270 kDa.^43^ Citric acid (99%), sodium hydroxide (NaOH), lysine (LYS), betaine (BET), L-cysteine (CYS), sodium hydroxide, hydrochloric acid, nitric acid, hydrogen peroxide, sodium chloride (99%) and hydrogen tetrachloroaurate trihydrate (99%) were supplied by Sigma-Aldrich. Silver nitrate (99%) was provided by Merck.

### 2.2 Fiber Preparation

All of the polymers were dissolved in distilled water at 70 °C in 20 mL sealed glass vials. Table 1 summarizes the compositions of the different polymer solutions. Citric acid, used as an in situ cross-linking agent, was added to all the blends at 15 wt % of the total polymer weight. BET, LYS, and CYS were added at a concentration of 10 wt % to GLU2. This amount was chosen based on previous research conducted by our group,^71,72^ which indicated that 10 wt % is the optimal concentration for achieving the desired functionalization of the spun mat. The electrospinning setup was self-made and is composed of a 3 mL plastic syringe coupled with a pump, a high-voltage power supply, and a stainless-steel collector. The electrospinning process was performed using the conditions optimized in the previously mentioned research: 30 kV field strength, 15 cm tip-to-collector distance, and a flow rate of 1.2 mL h^–1^. After electrospinning, fibers were cured by thermal treatment at 180 °C for 30 min.

**Table 1:** Prepared Fiber

Sample Name	Amount in GLU2 blend (wt %)	Amount in citric acid (wt %)^a^
GLU2	0	15
GLU2-BET	10% BET	
GLU2-LYS	10% LYS	
GLU2-CYS	10% CYS	

a Calculated based on the total weight of the polymer blend.

### 2.3 Characterization

The morphology of the fibers was examined by using scanning electron microscopy (SEM) and atomic force microscopy (AFM). SEM images were obtained using a Tescan VEGA 3 instrument (Brno, Czech Republic), operating at an accelerating voltage of 15 kV, a working distance of 10 mm, and a beam current of 100 pA. To prevent sample charging during SEM analysis, samples were first coated with a gold thin film, 15 nm in thickness, using a Vac Coat DSR1 sputter coater (London, UK), which operated at 60 mA. The mean fiber diameter was determined using ImageJ software, based on 100 measurements for each sample. AFM images were collected using a Nanosurf Easyscan2 instrument, which was placed in an insulated enclosure and positioned on an antivibration platform to minimize external mechanical and electrical perturbations. The instrument was equipped with a scanner capable of operating in the 70 × 70 × 12 μm (X × Y × Z) range. Measurements were conducted in air at room temperature using the intermittent contact mode, with the cantilever excited to oscillate near its resonance frequency of approximately 190 kHz. Sharp Si probes (Tap190Al-G, BudgetSensors) were used, with cantilever lengths of 225 μm, tip heights of 17 μm, and a probe apex radius of ≤10 nm. AFM images were recorded with a resolution of 256 × 256 pixels at a scan rate of 0.15–0.2 Hz. A PerkinElmer Spectrum 100 FTIR Spectrometer (Waltham, MA, USA) equipped with a universal ATR (attenuated total reflection) sampling accessory was used for recording FTIR spectra. The spectra were collected in the wavenumber range from 650 to 4000 cm^–1^, with a resolution of 4 cm^–1^. Thermogravimetric analysis was performed by using the TA Q500 instrument (New Castle, USA) to evaluate the thermal stability of the fibers. The initial sample weight was around 10 mg. Measurements were carried out in a nitrogen atmosphere, over a temperature range of 30 to 700 °C, with a heating rate of 10 °C min^–1^. The chemical composition of the fibers (CHNS elemental analysis) before and after washing was measured by using a Thermo Fisher FlashEA 1112 Series elemental analyzer (Waltham, MA, USA).

The pH of zero charge (pH_pzc_) was determined using the procedure reported by Faria et al.:^4^ 25 mg of the material was introduced into six vials containing 5 mL of NaCl (0.01 mol L^–1^) then the pH was then adjusted in the range of 2 to 12 using HCl or NaOH (0.1 and 1 mol L^–1^). The mixtures were maintained under stirring for 48 h. Finally, the final pH was measured using a pH meter (Metrohm, Switzerland). The pHpzc is the intersection point of the graph of Initial pH-Final pH versus Initial pH with the *x*-axis. The solubility test was performed using the protocol reported by Cecone et al.^66^ Briefly, 50 mg of the material was placed into 5 mL plastic containers. Then, 5 mL of distilled water was added, and the samples were left at room temperature for 24 h. After centrifugation, the fibers were dried in an oven at 60 °C until they reached a constant weight. Each test was performed in triplicate. The soluble fraction was calculated using the following equation:

1 $$\mathrm{Soluble fraction}\;(\%)=\frac{\left(W_{\mathrm{ini}}-W_{\mathrm{fin}}\right)}{W_{\mathrm{ini}}}\times 100$$

Where *W*_ini_ is the initial weight of the fibers (mg), and *W*_fin_ is the weight (mg) after drying.

### 2.4 Adsorption Tests

The adsorption of Au and Ag nanoparticles was performed using 10 mg of fibers in 5 mL of nanoparticle suspension at a pH of 6.80 for 24 h (200 rpm). AuNPs were prepared using the trisodium citrate reduction method according to the procedure proposed by Li et al.^73^ Briefly, 2 mL of chloroauric acid solution (25 mM) was introduced into a flask, followed by 6.6 mL of NaOH (20 mmol L^–1^) and distilled water to reach a final volume of 20 mL. After boiling this mixture for 30 min, 0.6 mL of sodium citrate solution (50 mg mL^–1^) was introduced rapidly under vigorous stirring and kept boiling for 2 min. The solution color changed from yellow to ruby red. The suspension was then cooled and diluted to 100 mL. AgNPs were synthesized according to the procedure reported by Oprica et al.^74^

The dried fibers were digested, and the amounts of Au and Ag were determined by ICP-OES measurements. The samples were digested in a microwave oven (Milestone-MEGA 1200). In the case of Au, a mixture of 3 mL of nitric acid and 1 mL of hydrogen peroxide in 100 mL tetrafluoromethoxyl vessels was added to the fibers. For Ag, the mixture was composed of 6 mL of hydrochloric acid, 1 mL of nitric acid, and 3 mL of nitric acid. The digestion program was as follows: 5 min at 250 W, 5 min at 400 W, 5 min at 600 W, 5 min at 250 W, and finally 30 min of ventilation. The resulting solutions were filtered through cellulose filters with a pore size of 0.45 μm (Whatman grade 5) to eliminate the undissolved polymer parts and diluted to 10 mL with ultrapure water (Milli-Q (Millipore), resistivity = 18.2 MΩ·cm). Au and Ag concentrations were determined by Inductively Coupled Plasma Optical Emission Spectroscopy, ICP-OES (PerkinElmer, model Optima 7000 DV), equipped with a PEEK Mira Mist nebulizer, a cyclonic spray chamber, and an Echelle monochromator. The radiofrequency power applied was 1300 W. Plasma, auxiliary, and nebulizer gas flows were 15, 0.2, and 0.6 L min^–1,^ respectively. The signals were measured in triplicate. The Au and Ag concentrations were measured at 267.595 nm and 328.068 nm, respectively.

The efficiency of GLU2-based fibers toward crystal violet (CV) removal was evaluated in a batch adsorption study. The experimental conditions used during the adsorption tests were a CV concentration of 50 mg L^–1^, pH equal to 5.88, 1 g L^–1^ fibers, and stirring at 450 rpm (tested volume = 10 mL) for 24 h. The residual concentration of CV was determined using a Varian Cary 300 Scan UV–visible spectrophotometer, measuring the absorption at 590 nm after the removal of the fibers.

These fibers were also tested for atenolol (ATN) removal from water. Batch adsorption tests were conducted using 10 mg L^–1^ ATN at room temperature, a pH value of 6.88, and an adsorbent amount of 1 g L^–1^. The concentration of ATN was determined using the method reported by Cecone et al.^66^ The instrument used was a Dionex (Sunnyvale, CA, USA), composed of a P680 pump and a UVD170U detector UVD170U. Separation was achieved using a Phenomenex (Torrance, CA, USA) Kinetex C18 (150 mm × 4.6 mm × 5 μm) column, and the mobile phase consisted of 0.1% orthophosphoric acid buffer and acetonitrile in a ratio of 90:10 v/v. The mobile phase was filtered by using a 0.45 μm nylon filter and degassed before use. The run time for the experiment was 5 min, with a retention time of 3.5 min for ATN and a flow rate of 1 mL min^–1^. The quantification of ATN was performed at 230 nm, and the detection limit was 0.1 mg L^–1^.

The removal percentage *Y* (%) of each pollutant was calculated using the following equation:

2 $$Y=\frac{\left(C_{0}-C_{e}\right)}{C_{0}}\times \;100$$

where *C*_0_ (mg L^–1^) is the initial concentration, *C*_e_ (mg L^–1^) is the concentration at equilibrium.

All presented data were obtained in triplicate and expressed as the mean ± standard deviation (SD). The ANOVA test was used to study the differences in the efficiencies of the four fibers toward the studied pollutants. The p, F, and F-critical values were calculated for the four fibers based on three replicates. The null hypothesis assumes no significant difference among the groups. If the observed F-value is greater than the F-critical value and the p-value is less than 0.05, the null hypothesis is rejected. This indicates that there is a statistically significant difference among the group means.

## 3 Results and Discussion

### 3.1 Physico-Chemical Characterization of the Fibers

CHNS elemental analysis of the different fiber results is presented in Table 2. The presence of nitrogen in the different mats was confirmed after the addition of BET, LYS, and CYS. However, the percentage of nitrogen decreased after washing, indicating the release of un-cross-linked compounds. The final amount after washing was higher for LYS and CYS than for BET. This can be attributed to cross-linking in the presence of amino acids through the formation of amide bonds, as described later in the FTIR analysis. The solubility test shows that more than 80 wt % of the initial material is insoluble for all the fibers, as shown in Figure S1. GLU2 exhibits a lower soluble fraction (5%), while higher percentages were observed after the addition of BET, LYS, and CYS. The observed weight loss can be associated with uncross-linked maltodextrin and the added compounds. Figure S2 presents the pH of zero charge (pH_pzc_) of the GLU2-based fibers. This parameter is fundamental to understanding the total surface charge of materials depending on the solution pH. If the pH is lower than pH_pzc_, then the membrane will be positively charged. If the solution pH is higher, the surface charge will be negative. They were equal to 2.74, 2.11, and 2.24 for GLU2, GLU2-BET, and GLU2-LYS, respectively. GLU2-CYS presents a pH of zero charge higher than the other fibers, reaching 3.60.

**Table 2:** Elemental Analysis of the Electrospun Fibers

Fibers composition^a^		%N	%C	%H	%S
GLU2	BW	0.00 ± 0.00	39.57 ± 0.03	5.99 ± 0.11	0.00 ± 0.00
	AW	0.00 ± 0.00	38.86 ± 0.05	5.66 ± 0.07	0.00 ± 0.00
GLU2-BET	BW	0.82 ± 0.01	40.64 ± 0.14	5.88 ± 0.06	0.00 ± 0.00
	AW	0.23 ± 0.10	39.00 ± 0.13	5.75 ± 0.09	0.00 ± 0.00
GLU2-LYS	BW	1.38 ± 0.03	43.29 ± 0.05	5.78 ± 0.24	0.00 ± 0.00
	AW	0.77 ± 0.02	41.46 ± 0.30	5.78 ± 0.04	0.00 ± 0.00
GLU2-CYS	BW	0.92 ± 0.02	40.10 ± 1.05	5.85 ± 0.18	1.80 ± 0.11
	AW	0.57 ± 0.02	39.44 ± 0.28	6.05 ± 0.01	0.69 ± 0.04

a BW: fibers before washing; AW: fibers after washing.

The morphology of the fibers was investigated by SEM analysis. As illustrated in Figure 1, the three mats derived from GLU2 displayed fibrous morphology, even following thermal cross-linking. High-molecular-weight polysaccharides, such as GLU2, facilitate the formation of stable jets and enable fiber production from all blends during electrospinning processes.^61^ The various mats exhibited a smooth surface, suggesting good miscibility of GLU2 with BET, LYS, and CYS.^75^ All of the samples displayed bead-free fibers, indicating that the electrospinning parameters and polymer concentration were optimized effectively. The incorporation of CYS and BET in the chosen ratio does not adversely affect the resulting fibrous morphology. However, a larger size distribution for GLU2-LYS fibers was observed (Figure 1E,F). Bonakdar et al. highlight that a stable Taylor cone is essential for producing uniform nanofiber diameters, while instability can cause irregular fiber distribution.^76^

**Figure 1:**
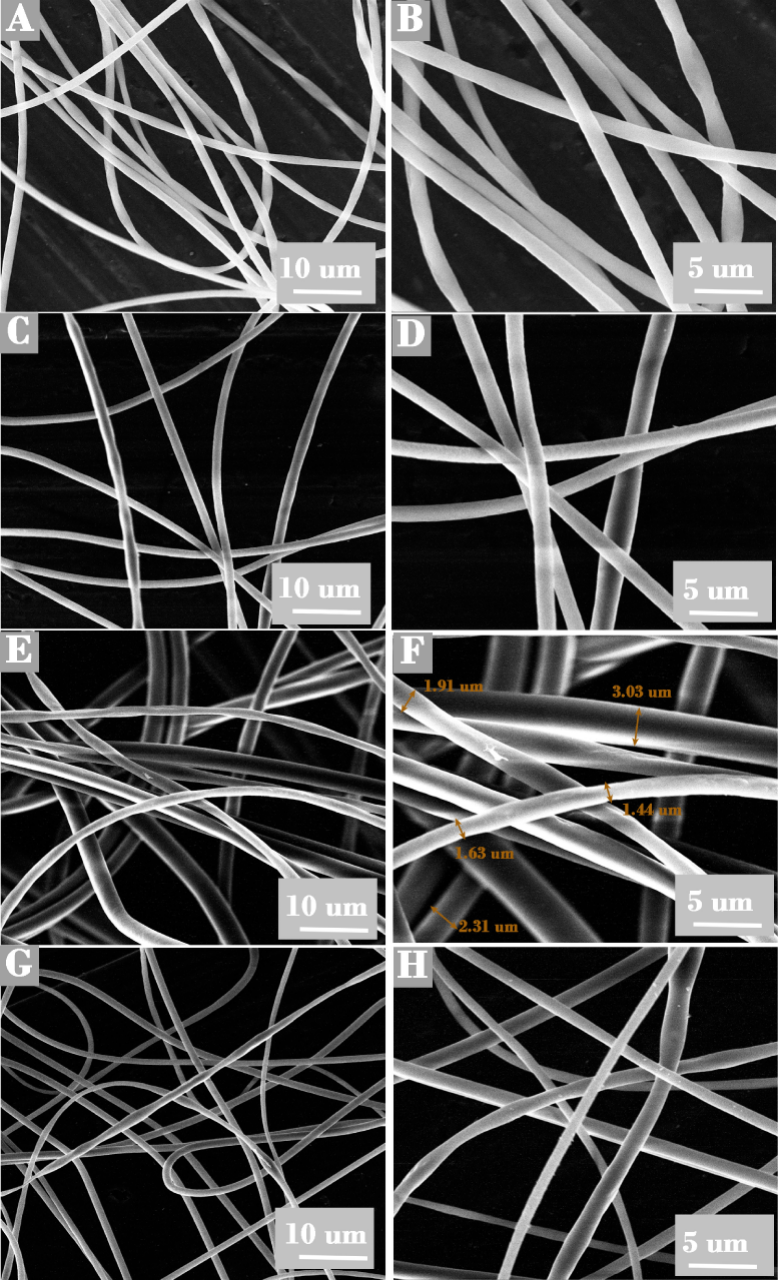
SEM images of thermally cross-linked electrospun fibers: GLU2 (A, B), GLU2-BET (C, D), GLU2-LYS (E, F), and GLU2-CYS (G, H)

The diameter of the fibers can be influenced by several parameters related to the properties of the blend during electrospinning. These parameters include changes in surface tension, viscosity, conductivity, and polymer concentration. Figure 2 illustrates the diameters of the various prepared fibers. Pristine GLU2 fibers have an average diameter of 1.28 ± 0.23 μm, which is consistent with previous studies on electrospun maltodextrin fibers with a dextrose equivalent of 2.^43^ An increase in diameter was observed for the GLU2-BET and GLU2-LYS samples, measuring 1.50 ± 0.23 μm and 2.16 ± 0.53 μm, respectively. Conversely, thinner fibers, measuring 1.18 ± 0.20 μm, were produced with the addition of CYS. The largest diameter of the fibers was observed when lysine was incorporated. The amino and carboxyl groups in lysine are likely to form hydrogen bonds with the hydroxyl groups of maltodextrin and water molecules. This interaction enhances the bonding between the polymer chains, leading to the formation of larger fibers.^61,77^ In contrast, the smaller diameter observed in GLU2-BET compared to GLU2-LYS can be attributed to the increase in solution conductivity following the addition of betaine as a hydrochloride salt.^78,79^ Smaller ions in the electrospinning solution typically lead to the formation of thinner fibers because smaller ions exhibit higher mobility in an electric field, resulting in greater elongation of the electrospinning jet and ultimately producing thinner fibers.^80,81^ In the case of GLU2-CYS, the observed thinning effect may be attributed to the presence of a less polar thiol group. This characteristic may be associated with a reduction in surface tension, leading to the formation of thinner fibers.^81^

**Figure 2:**
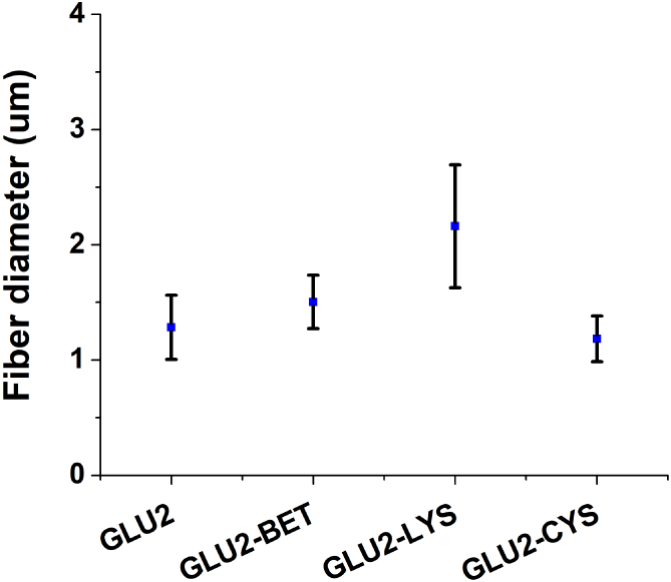
Size distribution of the fibers.

Figure 3 presents the 3D and 2D AFM topographic images of thermally cross-linked electrospun fibers, including GLU2 (A, A’), GLU2-BET (B, B’), GLU2-LYS (C, C’), and GLU2-CYS (D, D’).

**Figure 3:**
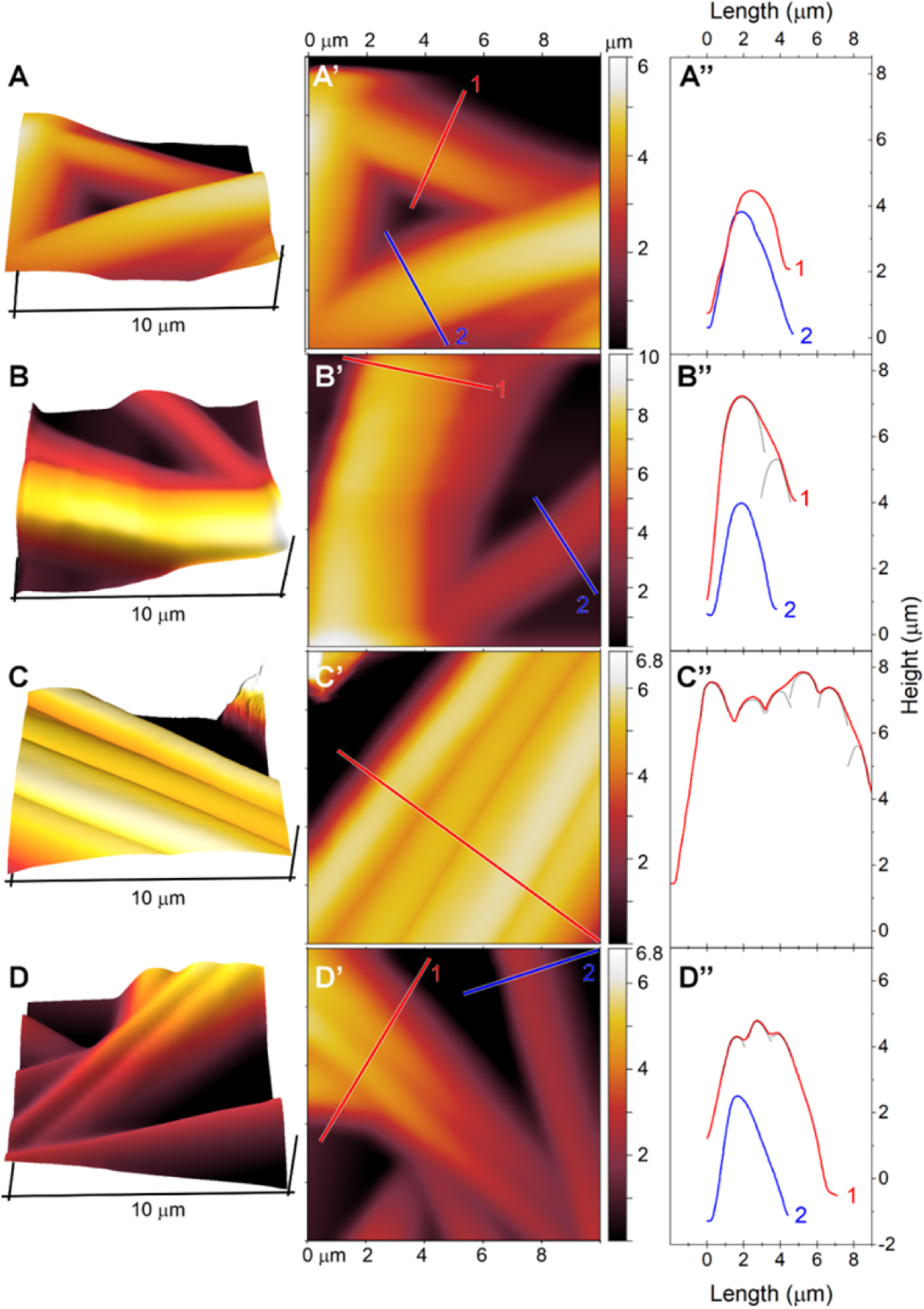
3D and 2D 10 × 10 μm AFM topographic images of thermally cross-linked electrospun fibers: GLU2 (A, A’), GLU2-BET (B, B’), GLU2-LYS (C, C’), and GLU2-CYS (D, D’). In A”, B”, C”, and D”, the corresponding height profiles along selected lines are shown, respectively.

The 3D AFM images clearly illustrate the three-dimensional morphology of the fiber mats, revealing a generally smooth and uniform surface morphology across all samples. However, there are remarkable differences in surface properties among the pristine (GLU2) and functionalized fibers (GLU2-BET, GLU2-CYS, and GLU2-LYS). These fibers appear locally entangled and randomly distributed, forming porous textures (GLU2, GLU2-BET, and GLU2-CYS) or locally with more bundled arrangements (GLU2-LYS). The 2D AFM topographic images (Figure 3A’,B’,C’,D’), along with the related height line profiles (Figure 3A”,B”,C”,D”), indicate that the fibers have different diameters, but all fall within the 1–3 μm diameter range. The observed overall fiber diameters can be higher due to the entanglement of the different fibers (see dotted line profiles in Figure 3B”,C”,D”). The height profiles from selected lines further confirm that the fibers are well-rounded and have smooth surfaces. This smooth surface texture, observed in the AFM images obtained at a higher resolution and more localized scale (10 μm × 10 μm), corresponds well with SEM observations obtained at lower magnification (Figure 3B,D,F,H). AFM imaging reveals a very smooth fiber surface, which, combined with FTIR analysis (Figure 4), confirms a strong affinity between maltodextrin and functional additives. This is likely due to interactions between the high density of hydroxyl (−OH) groups on maltodextrin and the amino (−NH_2_) and carboxyl (−COOH) groups on the additives, all of which are highly polar and hydrophilic. In summary, the variations in surface texture and fiber arrangement reflect the impact of different cross-linking agents on the fiber microstructure.

**Figure 4:**
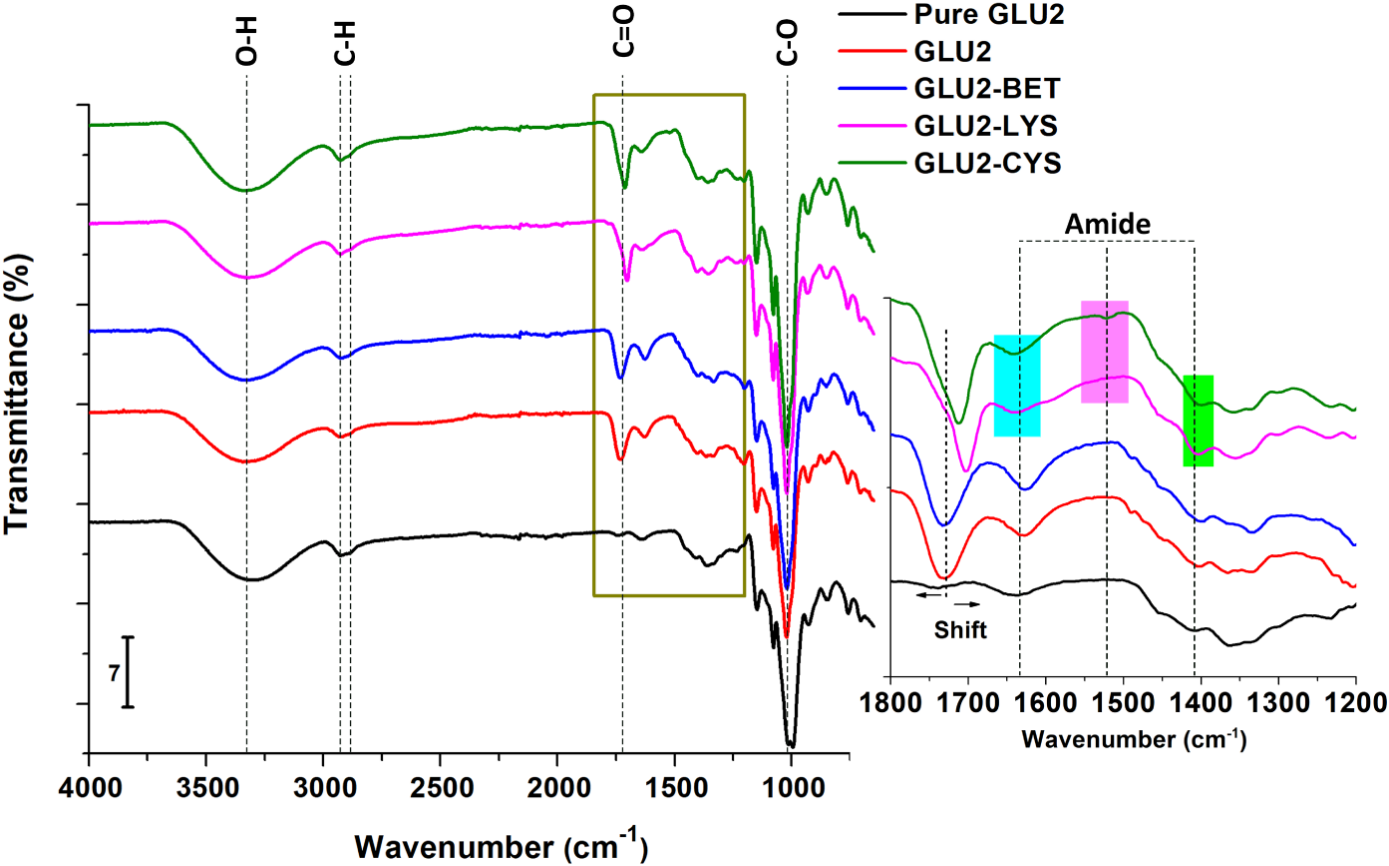
FTIR spectra of pure GLU2, GLU2-BET, GLU2-LYS, and GLU2-CYS (detailed signals in the range of 1400–1800 cm^–1^: Blue band 1600–1680 cm^–1^, Pink band 1490–1550 cm^–1^, Green band 1400–1410 cm^–1^).

Figure 4 presents the FTIR spectra of pure GLU2, GLU2-BET, GLU2-LYS, and GLU2-CYS. The characteristic signals of maltodextrin were detected in all of the samples. The strong absorption band between 3000 and 3600 cm^–1^ corresponds to the stretching vibration of the hydroxyl group O–H of carbohydrates.^82^ The signal observed at 2922 cm^–1^, is assigned to C–H stretching.^58^ The peaks at 1422 and 1366 cm^–1^ are related to the deformation vibration of C–H in the −CH_2_ and −CH_3_ bonds.^83^ Kačuráková et al. reported that the stretching vibrations of the C–O–C glycosidic bridge appear in the spectral ranges of 1160–1130 and 999–965 cm^–1^.^84^ All the GLU2-based fibers present a broad band around 1150 and 980 cm^–1^, corresponding to the combination of the vibration of the C–C pyranoid ring of the maltodextrin’s glucose monomers, the vibration of the C–O–C glycosidic bond, and the stretching of the C–OH side group.^58^ Compared to the pure maltodextrin spectrum, a new band appears at 1724 cm^–1^ in the GLU2 cross-linked with citric acid. This band is correlated to the carbonyl C=O of the ester group formed during the thermal treatment. This esterification occurs through a condensation reaction between the carboxylic acid groups of citric acid (CA) and the hydroxyl groups of maltodextrin.^85^ After the addition of BET, a shift of this band to a higher wavelength (1733 cm^–1^) is observed. However, this band shifts to lower wavelengths, 1703 cm^–1^ for LYS and 1713 cm^–1^ for CYS, respectively. The band shifting confirms the change in the chemical environment and the presence of new interactions due to the addition of BET, LYS, and CYS in the fibers.^72^

In the case of amino acid addition to maltodextrin, the Maillard reaction can occur during the thermal treatment of the mats.^62^ This reaction starts with the condensation between the carbonyl groups of polysaccharides and the free amino groups of amino acids, peptides, and proteins, forming an unstable Schiff base. This base then undergoes an Amadori rearrangement to form reactive α-carbonyl species capable of reacting with additional nucleophiles, such as other amines. Finally, when the polysaccharide reacts with amino groups, advanced glycation end products are formed, and low- or high-molecular-mass products, called melanoidins, are generated.^86–88^ The cross-linking through the Maillard reaction was confirmed by the characteristic bands of amide groups in the regions 1600–1680 cm^–1^ (blue band), 1490–1550 cm^–1^ (pink band), and 1400–1410 cm^–1^ (green band) for GLU2-CYS and GLU2-LYS FTIR spectra. The amide I signal appearing at 1641 cm^–1^ is related to the stretching vibrations of the C=O and C–N groups.^58^ The amide II band around 1522 cm^–1^ and the amide III band observed at 1405 cm^–1^ are correlated to N–H bending^71^ and C–N stretching,^89^ respectively.

The effect of adding BET, LYS, and CYS on the thermal properties of electrospun GLU2-based fibers was investigated by using TGA. Figure 5 displays the TGA curves of the electrospun mats along with their corresponding differential thermogravimetric analysis (DTGA) curves. All the fibers demonstrated thermal stability up to 200 °C. They exhibited a first weight loss, attributed to water evaporation, ranging from 4% to 8% by weight.^37^ A two-stage degradation profile was observed for GLU2, while GLU2-BET, GLU2-LYS, and GLU2-CYS exhibited a three-stage degradation profile. The maltodextrin degradation occurred in a temperature range of 200 to 350 °C, which involved the breakdown of glycosidic bonds and the release of volatile compounds.^62,66^ Karaaslan et al. indicated that polysaccharides began to decompose rapidly at temperatures around 300 °C.^90^ Upon adding betaine, the TGA profile showed a similar trend; however, the total weight loss during the two stages was 6% lower compared with the maltodextrin fibers. Additionally, the carbon residue at 700 °C increased from 12% to 18%. While the temperature corresponding to the maximum degradation rate was not significantly different for GLU2 and GLU2-BET (305 and 307 °C, respectively), it slightly shifted to lower values with the addition of amino acids, reaching 287 °C for GLU-LYS and 288 °C for GLU-CYS. The highest carbon residue observed was after the addition of lysine, equal to 20% at 700 °C.^88^ The DTGA curves for GLU2-LYS, and GLU2-CYS displayed a third weight loss around 400 to 500 °C, which was absent in the GLU2 profile. The presence of this weight loss indicates the stabilization of the pyrolysis products because of the Maillard reaction taking place between the amino acids and maltodextrin, as previously demonstrated in the FTIR analysis (Figure 5).^91^

**Figure 5:**
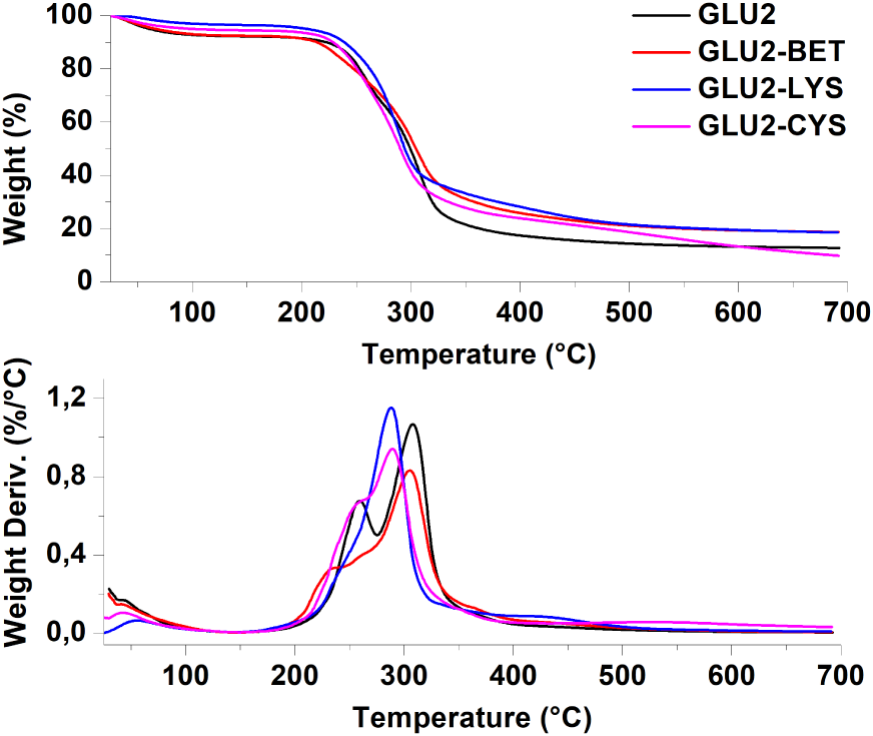
TGA and related DTGA of GLU2-based fibers.

### 3.2 Application in Nanoparticle Removal

This part focuses on the application of different maltodextrin-based fibers as sustainable adsorbents for metallic nanoparticles. AgNPs, synthesized according to the procedure reported by Oprica et al.,^74^ were characterized using UV–visible spectroscopy, TEM, and zeta potential measurements. Results are listed in Figure 6. TEM images (Figure 6A,B) demonstrated particles with different morphologies, including rods, spheres, and hexagonal nanoplates, and a broad size distribution, as described by Zhang et al.^92^ Figure 6A,B also shows a stable dispersion of AgNPs without the formation of aggregates, attributed to the presence of the capping agent.^93^ The UV–visible spectrum of the nanoparticles presents a typical surface plasmon resonance absorption band with a maximum at 430 nm.^94^ The zeta potential graph (Figure 6D) proves that AgNP dispersion is stable for pH values higher than 5, with a zeta potential reaching −60 mV around pH 8 to 10. This negative value could be explained by the surface adsorption of citrate ions. However, the suspension remains unstable around the pH of zero charge (3.86), with eventual aggregation of the particles.

**Figure 6:**
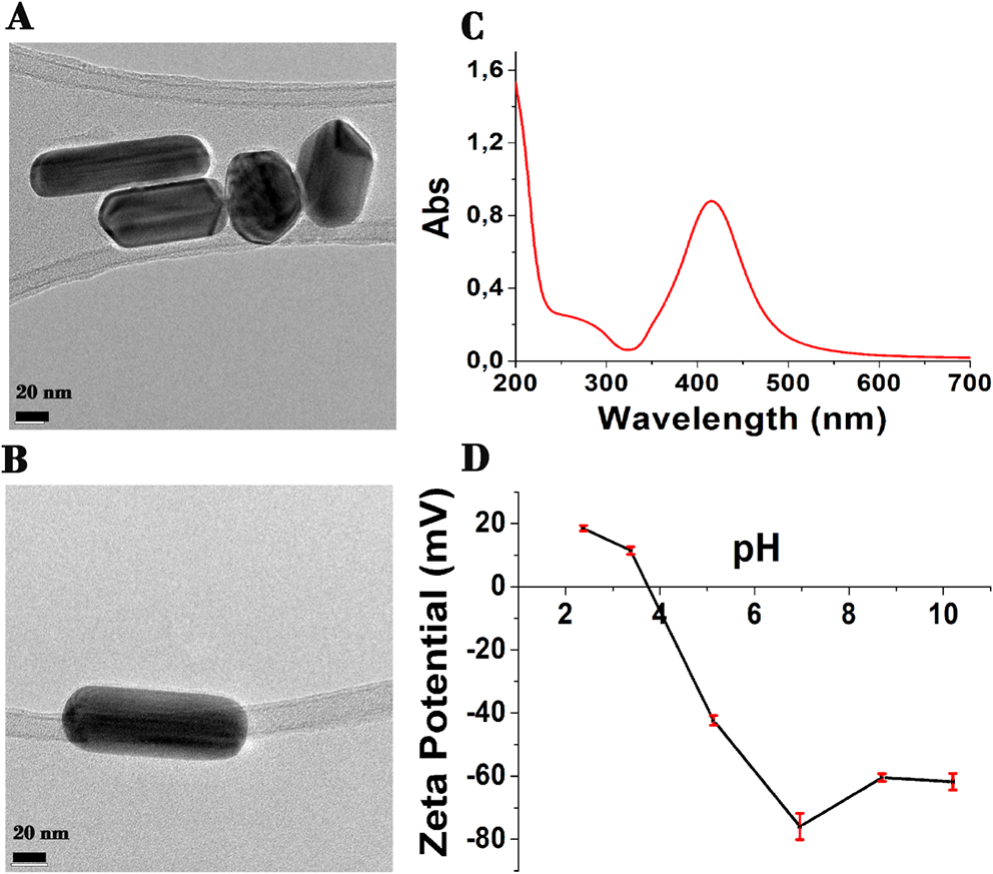
TEM images of AgNPs (A, B), UV–visible spectrum of AgNPs (C), and zeta potential of AgNPs (D).

The efficiency of GLU2-based fibers toward AgNPs adsorption was confirmed by digestion of the fibers and ICP-OES measurements of Ag following the adsorption experiments. Table 3 presents the concentrations of AgNPs adsorbed onto the fibers.

**Table 3:** Concentrations of Adsorbed AgNPs

Fibers	[AgNPs] (mg L^–1^)
GLU2	0.06 ± 0.01
GLU2-BET	0.23 ± 0.05
GLU2-LYS	0.12 ± 0.02
GLU2-CYS	0.49 ± 0.07

The UV–visible spectra of the AgNPs solutions were also recorded after adsorption tests, as shown in Figure 7. The results obtained from the digestion of different fibers show a low adsorption capacity of silver nanoparticles. However, UV–visible analysis demonstrated a higher removal efficiency for some fiber mats. This apparent inconsistency between the results may be partially attributed to the hypothesis of the aggregation of these nanoparticles during the experiments. While we acknowledge that this interpretation should be strengthened by additional characterization in future work, visual evidence supports this possibility. As shown in Figure S3, the glass tube used in the adsorption tests displays a visible gray ring, which is consistent with AgNP aggregation and sedimentation. Silver nanoparticles are known to be sensitive to surface chemistry and environmental conditions.^95^ Lodeiro et al. demonstrated that polysaccharide-coated AgNPs can exhibit dramatically different aggregation kinetics in saline solutions.^96^ Nanoparticle aggregation leads to a reduction in the effective surface area and thus lower adsorption capacities. This is further demonstrated by recent literature, such as the work by Jian et al., who demonstrated that the aggregation behavior of nanosized alumina was a critical factor for the adsorption of PFOS, directly linking increased aggregation to decreased adsorption efficiency.^97^ Gilbert et al. demonstrated that ferrihydrite nanoparticle aggregation reduces copper adsorption, highlighting how different aggregation processes can lead to varied aggregate structures that subsequently impact ion uptake.^98^

**Figure 7:**
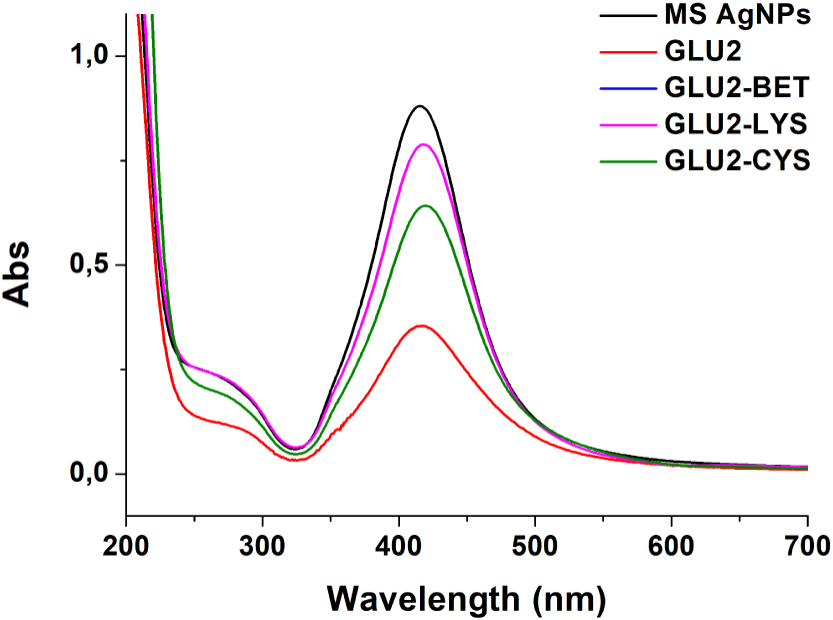
UV–visible spectra of AgNP solutions after adsorption.

The prepared GLU2-based fibers were also tested as adsorbents for gold nanoparticles. Synthesized AuNPs were characterized by using UV–visible spectroscopy, TEM, and zeta-potential methods. The UV–visible spectrum (Figure 8A) shows that the surface plasmon resonance band occurs at 520 nm, confirming the successful preparation of AuNPs. TEM images (Figure 8B) show the spherical shape of the nanoparticles, with an average size of around 13 ± 2 nm (Figure 8C). Zeta potential measurements were all negative for the pH range of 3 to 10, as presented in Figure 8D, indicating good stability of the suspension. The adsorption tests on the different fibers were monitored by using the digestion of the dried membranes and ICP measurements of Au adsorbed on these fibers, as reported in Table 4. The statistical tool ANOVA test was applied to compare the efficiency of these fibers (Table S1). The results of this test show a p-value of less than 0.05 and an F-value of 21.26, which is higher than the F-critical. This indicates that there is a significant difference between the adsorption abilities of the four fibers at a confidence level of 95%. GLU2-LYS is the most effective fiber for gold nanoparticle remediation, followed by GLU2, whereas GLU2-BET and GLU2-CYS are less suitable due to their poor retention capabilities. The GLU2-LYS fibers present a pH of zero charge equal to 2.24, indicating that the fiber surface carries a negative charge at an experimental pH of 6.80. Since citrate-capped gold nanoparticles (AuNPs) also possess a negative surface charge, strong electrostatic repulsion would be expected between the fibers and the AuNPs. Nevertheless, the inductively coupled plasma (ICP) analysis revealed a desorbed AuNP concentration of 9.42 mg L^–1^ (Table 4), indicating significant adsorption of AuNPs onto the fibers despite the electrostatic barrier. These results support the conclusion that the coordination between nitrogen atoms of lysine’s α-amino groups and gold atoms dominates the adsorption mechanism, overcoming the electrostatic repulsion. This is consistent with the NMR and spectroscopic investigations demonstrated by Selvakannan et al.^99^ and confirms that amine groups in lysine strongly bind AuNPs. Similar findings were reported in the study of lysine-functionalized PVA fibers for AuNP extraction from water.^72^ However, the incorporation of betaine and cysteine into maltodextrins resulted in significantly lower AuNP adsorption, with desorbed concentrations of 1.07 mg L^–1^ and 0.73 mg L^–1^, respectively. Both betaine- and cysteine-modified fibers exhibit low pH of zero charge values near 2, indicating that their surfaces are negatively charged. This surface charge results in electrostatic repulsion with the negatively charged citrate-capped gold nanoparticles, contributing to the observed lower adsorption affinity on these fibers compared with GLU2-LYS fibers. Thus, the reduced AuNP uptake by betaine- and cysteine-modified fibers can be attributed to this electrostatic repulsion, which is not sufficiently compensated by specific coordination or chemisorption mechanisms present in lysine-modified fibers.

**Figure 8:**
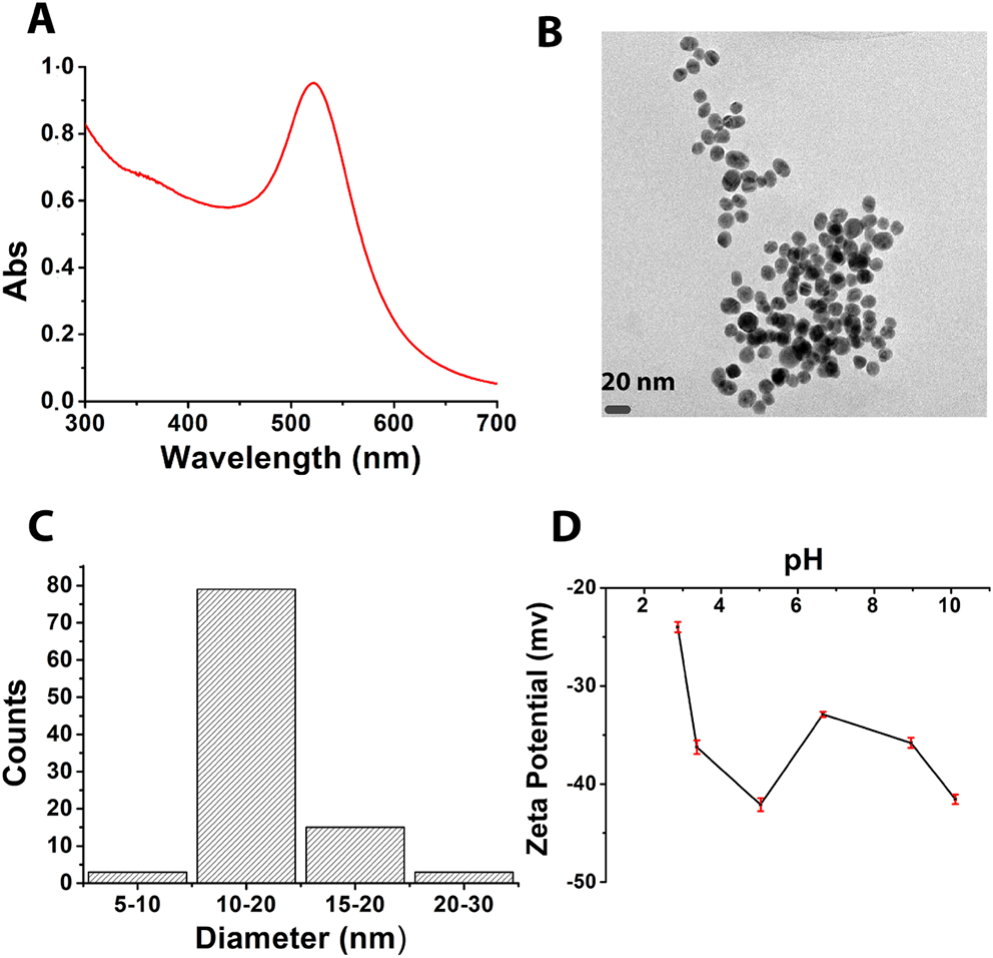
UV–visible spectrum of AuNPs (A), TEM image (B), size distribution of nanoparticles (C), and zeta potential of AuNPs (D).

**Table 4:** Concentrations of Adsorbed AuNPs

Fibers	[AuNPs] (mg L^–1^)
GLU2	5.06 ± 0.36
GLU2-BET	1.07 ± 0.12
GLU2-LYS	9.42 ± 3.03
GLU2-CYS	0.73 ± 0.19

### 3.3 Application in Atenolol Removal

Adsorption tests for ATN using the four prepared fibers were conducted at a concentration of 10 mg L^–1^ and a fiber dose of 1 g L^–1^. Figure 9 presents the removal efficiency of these materials. The unmodified GLU2 showed the highest removal efficiency at 81.73%, followed by GLU2-BET (68.68%) and GLU2-CYS (63.94%), while GLU2-LYS presented the lowest removal yield (41.91%). The ANOVA test was conducted to statistically validate these differences, as presented in Table S2. The p-value was much lower than 0.05, and the F-value (179.94) was greater than the F-critical value (4.06). The results indicate that the removal performance of the fibers is statistically significant and is in accordance with the adsorption efficiencies depicted in Figure 9. The effectiveness of these materials is influenced by several factors, including the pH of the solution, which affects both the ionization of atenolol and its interaction with adsorption sites.^100^ As reported in Figure S2, the pH of zero charge of the GLU2 fiber is around 2, indicating that the fiber surface carries a negative charge at the experimental solution pH of 6.88. Atenolol has a p*K*_a_ value of 9.43 (Figure S4), implying that the predominant form, at the tested pH is a positively charged cation due to the protonation of the amine groups.^101^ Favorable electrostatic attraction between ATN cations and the negatively charged surface of GLU2 promotes the adsorption of ATN onto the electrospun fiber surface. Similar electrostatic-driven adsorption behavior has been reported by Cecone et al. in their study on the surface modification of maltodextrin-based cross-linked electrospun mats targeting ATN removal.^71^ Despite the incorporation of lysine, betaine, and cysteine, these three fibers have shown lower removal efficiencies. Maltodextrin’s branched polysaccharide structure presents multiple hydroxyl groups capable of forming hydrogen bonds with polar molecules such as ATN, enhancing physical adsorption.^27^ However, the presence of lysine’s amine and carboxyl groups leads to a more positively charged fiber surface at neutral pH, which can reduce electrostatic attraction to cationic ATN. Betaine is zwitterionic and presents both positive and negative charges, reducing its overall affinity for ATN due to less favorable electrostatic or nonpolar interactions. L-cysteine’s thiol groups (−SH), although reactive, provide fewer adsorption sites compared to maltodextrin’s abundant hydroxyl groups and may contribute less to ATN binding. The combination of maltodextrins with these amino acids may compete for adsorption sites, reducing the overall efficiency of maltodextrin. Therefore, maltodextrin’s unique structural properties and functional groups make it a more effective adsorbent for atenolol.^102^

**Figure 9:**
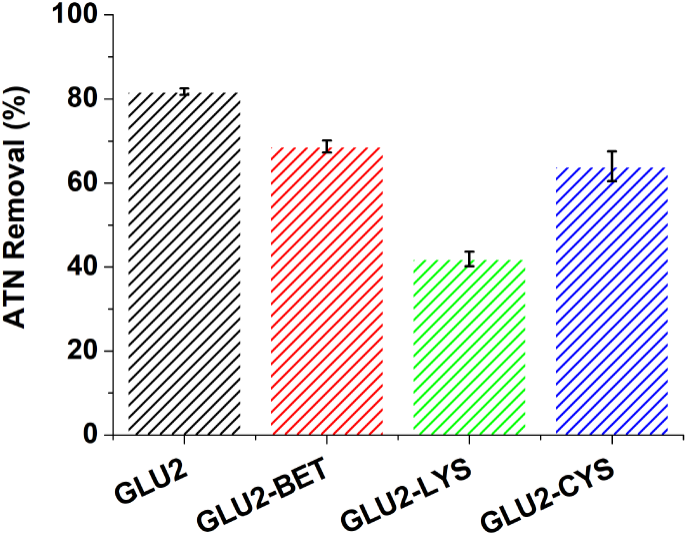
Adsorption tests of ATN. Experimental conditions: 1 g L^–1^ fibers, pH = 6.88, and 10 mg L^–1^.

### 3.4 Application in Crystal Violet Removal

Crystal Violet (CV) adsorption tests of the four prepared fibers were carried out at an initial concentration of 50 mg L^–1^. Figure 10 presents the efficiency of these fibers for CV abatement. GLU2-CYS fibers demonstrated the highest removal efficiency of 97.2% compared to the other three fibers, which showed removal yields between 40% and 47%. An ANOVA test was performed to check the differences between the four fibers regarding CV removal. Table S4 shows that the F-value was higher than the F critical and p-value was less than 0.05 for the three fibers GLU2, GLU2-BET, and GLU2-BET. This finding indicates that the null hypothesis is accepted and there is no difference between these three fibers. However, the comparison between the two groups, GLU2-BET and GLU2-CYS, demonstrates a significant difference in performance. The p-value is reported as less than 0.05, and the F-value (2950.17) greatly exceeds the F-critical value (7.71). These data indicate that the incorporation of cysteine significantly enhances CV adsorption. The enhancement is attributed to the introduction of functional groups such as thiol (−SH) and amino groups. The pH of zero charge after the addition of cysteine shifted higher to 3.60 (Figure S2), compared to a value of 2.00 for the other fibers. At the experimental pH of 5.76, the surface of GLU2-CYS is negatively charged but less so than the other fibers, favoring stronger electrostatic attraction with the positively charged quaternary ammonium groups of CV.^23,103^ Since the other fibers also presented a negatively charged surface under the experimental conditions but exhibited lower CV removal, electrostatic interactions alone do not explain the higher adsorption capacity. Hence, other physical interactions may be responsible for the superior adsorption on GLU2-CYS. Cysteine presents hydrophobic moieties and sulfur-containing groups that can interact with the aromatic rings of CV through π–π stacking and hydrophobic interactions, enhancing adsorption affinity.^104^ Similar findings were reported by Ben Khalifa et al. in their study on the surface modification of PVA fibers and their application for dye removal.^71^

**Figure 10:**
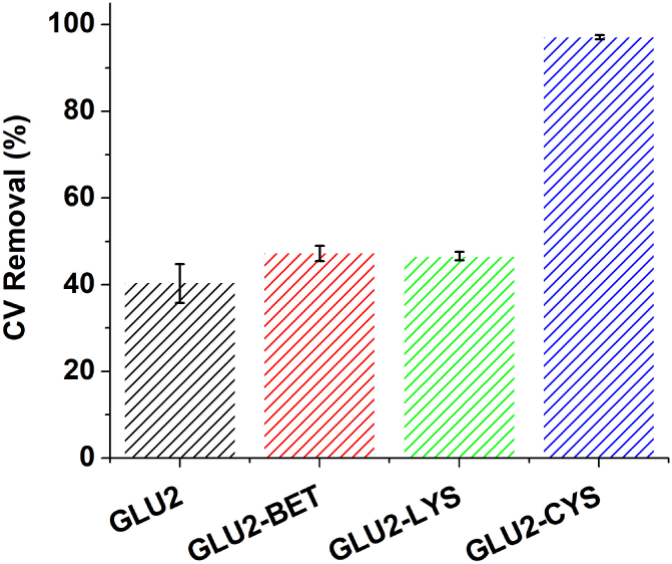
Adsorption tests of CV. Experimental conditions: 1 g L^–1^ fibers, pH = 5.76, and 50 mg L^–1^.

In comparison to other materials reported in the literature, GLU2-CYS achieves high removal efficiency under moderate conditions, without the need for elevated pH (5.76) and adsorbent amount (1 g L^–1^). Ba et al. reached 97.86% removal using calcium silicate waste at a higher adsorbent dose of 3.35 g L^–1^ and pH 6.87 ^105^ and Poornachandhra et al. achieved 94.75% removal with cellulose hydrogel at pH 9 and a 0.5 g dose.^106^ These comparisons illustrate the practical advantages and potential of GLU2-CYS fibers as an efficient and eco-friendly adsorbent for CV removal.

### 3.5 Conceptual Study of the Reusability of Fibers

The long-term utility of adsorbent materials in water remediation depends not only on their initial removal efficiency but also on their ability to withstand repeated use and perform under complex aqueous conditions. Although regeneration tests were not performed in this study, several of the properties observed in our maltodextrin-based fibers suggest promising potential for reuse.

The fibers prepared in this work, particularly those functionalized with lysine and cysteine, were thermally cross-linked through citric acid and Maillard-type reactions. This covalent stabilization was confirmed through FTIR and elemental analysis, and further supported by their high water insolubility (>80%) and preserved fibrous morphology after immersion. Specifically, solubility tests showed that all fibers remained mostly intact after 24 h in water, and the CHNS analysis confirmed that nitrogen and sulfur groups were retained after washing, particularly in the LYS and CYS samples, indicating the chemical stability of functional groups. In addition, the FTIR spectra revealed clear amide I, II, and III signals, particularly in GLU2-LYS and GLU2-CYS, consistent with the formation of covalent bonds between the maltodextrin backbone and amino acid additives. This chemical bonding provides resilience against leaching, which is critical for materials expected to undergo multiple adsorption/desorption cycles. Thermal analysis (TGA) showed all fibers were stable up to 200 °C, with additional degradation steps linked to Maillard-derived components, supporting structural integrity under mild regeneration or drying protocols.

The presence of a range of functional groups (−OH, −NH_2_, −SH, and −COOH) offers diverse pollutant interaction mechanisms, many of which are reversible. For instance, Cecone et al. reported that maltodextrin-based mats cross-linked with citric acid retained over 80% of their adsorption capacity for atenolol after three cycles using a mild acidic ethanol rinse.^66^ Similarly, Ruggeri et al. showed that amino acid-modified scaffolds prepared via Maillard-type cross-linking maintained more than 75% performance after five cycles of water–ethanol washing.^62^ These findings support the idea that fibers like ours, prepared via similar routes and exhibiting strong cross-linking, may be regenerable under mild and environmentally friendly conditions.

In real-world applications, wastewater rarely contains a single pollutant. Organic dyes, pharmaceuticals, heavy metals, and natural organic matter can all compete for adsorption sites. Previous studies have shown that this competition can reduce the overall capacity. For example, Zhu et al. found that dyes and heavy metals reduced each other’s adsorption by over 30% in binary systems due to competition for binding on negatively charged surfaces.^107^ Similarly, Rana et al. showed that cellulose-based hydrogels functionalized for multicontaminant adsorption still retained 60–70% efficiency despite these interactions.^108^

Despite these challenges, functionalized biopolymers, especially those with diverse chemical moieties, are often capable of synergistic binding. In our study, the wide variation in pollutant affinities among the different fiber formulations suggests that our materials can interact with multiple types of contaminants through various mechanisms. GLU2-CYS was highly effective for crystal violet (98%), likely due to thiol-aromatic interactions and electrostatic attraction. GLU2-LYS performed best for gold nanoparticles, probably through amine-metal coordination, while GLU2 fibers showed strong atenolol uptake, driven by electrostatic attraction and hydrogen bonding. Moreover, pHpzc analysis showed that GLU2 and its derivatives had surface charges well-suited for binding cationic pollutants at the tested pH levels, indicating that the electrostatic behavior would remain favorable even in complex matrices.

Taken together, the chemical functionality, structural resilience, and pollutant selectivity demonstrated in this work provide a strong basis for anticipating that these fibers can be reused and applied under realistic environmental conditions. Future work will be necessary to experimentally validate these projections through cyclic regeneration studies and mixed-pollutant tests, but the findings presented here offer a promising starting point.

## 4 Conclusion

In summary, this study investigated the incorporation of betaine, lysine, and cysteine into a maltodextrin mixture to prepare electrospun fibers with the appropriate functional groups necessary for effectively removing water pollutants. These fibers were characterized to assess their chemical compositions, functional groups, morphology, and thermal stability properties. Elemental analysis confirmed the presence of amino acids within the different electrospun fiber mats, as indicated by the increase in the percentages of nitrogen and sulfur. FTIR analysis demonstrated cross-linking through amide bonds after the addition of both amino acids, lysine and cysteine. The three maltodextrin-based fibers also maintained their fibrous morphology and smooth features. The average fiber diameter increased from 1.28 ± 0.23 μm (GLU2) to 1.50 ± 0.23 μm and 2.16 ± 0.53 μm for GLU2-BET and GLU2-LYS, respectively. However, the addition of cysteine led to the formation of the smallest average diameter, 1.18 ± 0.19 μm. AFM analysis confirmed the good affinity between maltodextrin and the added functional additives, which was attributed to the interactions between hydroxyl (−OH) groups on the maltodextrin and the amino (−NH_2_), carboxyl (−COOH), and sulfuryl (−SH) groups of the additives. The point of zero charge was also assessed to understand the adsorption behavior. Batch adsorption tests were performed on the four electrospun fibers to evaluate their efficiency as adsorbents of Ag and Au nanoparticles, atenolol, and crystal violet. GLU2-LYS exhibited the highest amount of adsorbed gold nanoparticles, reaching 9.42 mg L^–1^. None of the prepared fibers were efficient for silver nanoparticle removal from water due to their aggregation during the adsorption experiments. In the case of atenolol, GLU2 showed strong adsorption capabilities for the removal of this contaminant, achieving a removal percentage of 82%. The incorporation of the additives did not improve the adsorption capacity toward atenolol, as it reached 68% for GLU2-BET. GLU2-CYS fibers demonstrated remarkable efficiency in crystal violet removal, with the removal percentage increasing from 40% to 98%, after the incorporation of the amino acid.

These findings highlight the potential of amino acid-modified maltodextrin electrospun fibers for selective adsorption applications, especially for metal nanoparticles and organic pollutants. Future work could explore further chemical modifications or alternative additives to enhance the affinity and selectivity of these fibers for a broader range of pollutants, enabling their application in the environmental remediation field.

## Supplementary Material



## Data Availability

All data generated or analyzed during this study are included in this article.

## Notes

Soluble fraction of GLU2-based fibers (Figure S1); pH of zero charge of GLU2-based fibers (Figure S2); aggregation of AgNPs (Figure S3); ATN protonation equilibrium (Figure S4); ANOVA test results for AuNPs removal (Table S1); ANOVA test results for Atenolol removal (Table S2); ANOVA test results for CV removal (Table S3) (PDF)
