# Supplementary material for: Electrospun Maltodextrin Fibers for Efficient Removal of Nanoparticles, Atenolol, and Crystal Violet: Preparation and Characterization
Source: ACS Omega. 2025 Oct 15;10(42):49522–35. doi: 10.1021/acsomega.5c03266 (PMC12573020; doi:10.1021/acsomega.5c03266)
Supplement: Supplementary file 1 [file ao5c03266_si_001.pdf]

Electrospun Maltodextrin Fibers for Efficient Removal of Nanoparticles, Atenolol, and Crystal Violet: Preparation and Characterization

Eya Ben Khalifa[a], Claudio Cecone[a*], Boutheina Rzig[b], Giulia Mori[a], Federico Cesano[a], Mery Malandrino[a], Pierangiola Bracco[a], Giuliana Magnacca[a]

[a] Department of Chemistry and NIS Interdepartmental Centre, University of Turin, Via Pietro Giuria 7, 10125 Torino, Italy

[b] Ecochimie Laboratory, National Institute of Applied Sciences and Technology (INSAT), University of Carthage,1080 Tunis, Tunisia

Corresponding Author: Claudio Cecone, Email: claudio.cecone@unito.it

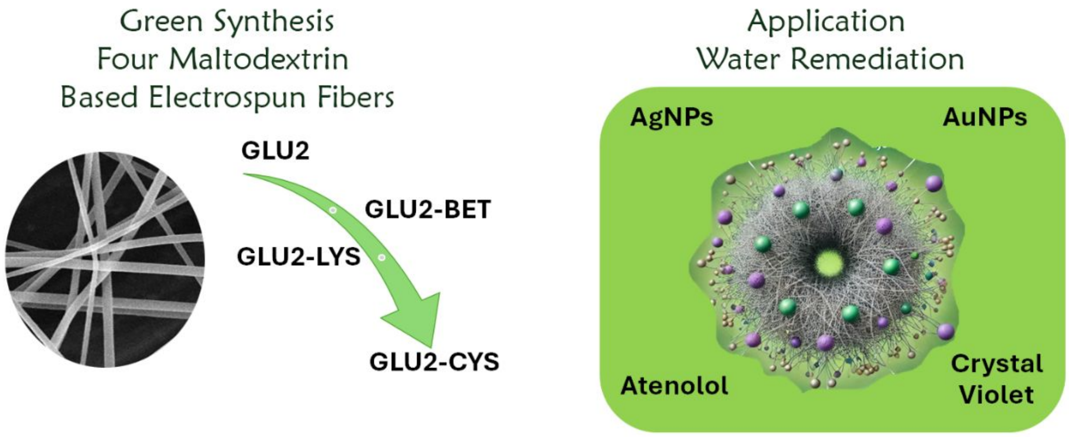

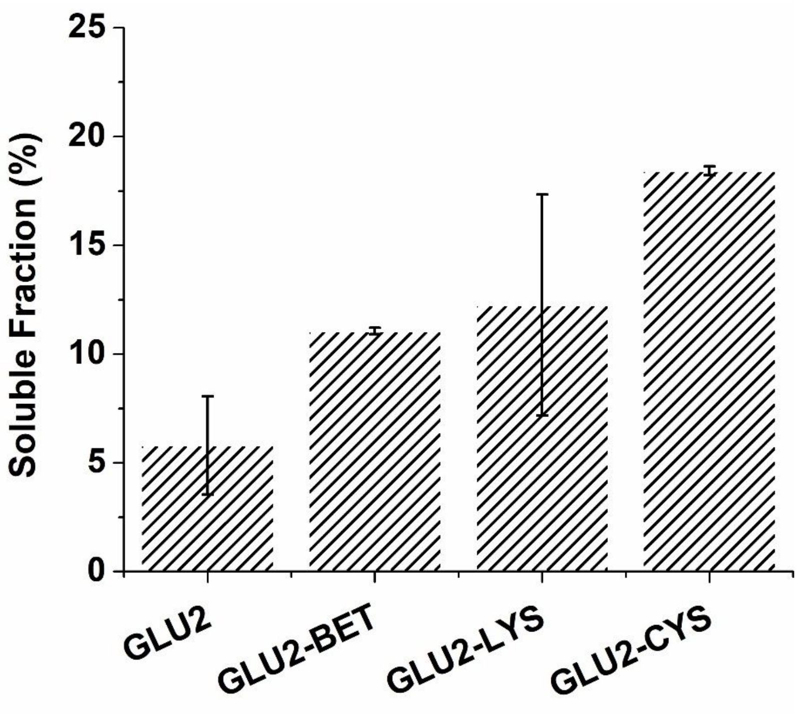

Fig. S1 Soluble fraction of GLU2 based fibers

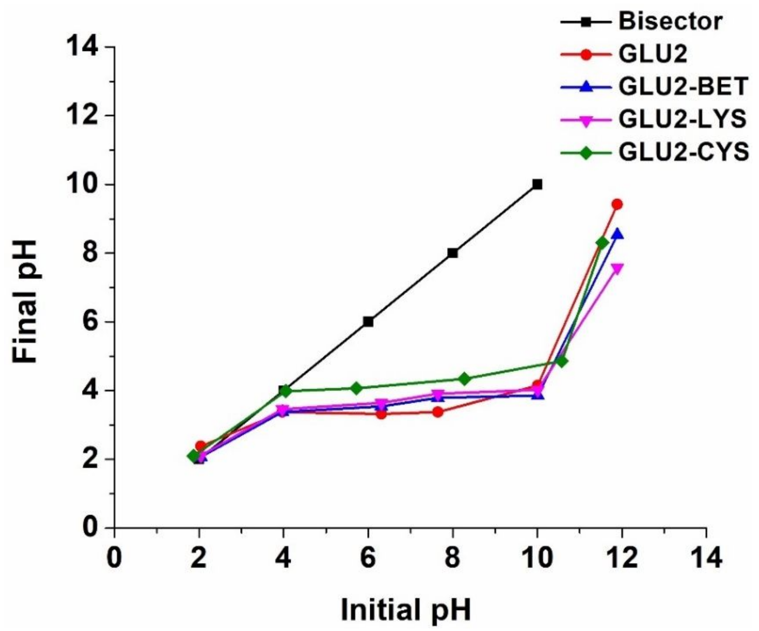

Fig. S2 pH of zero charge of GLU2-based fibers

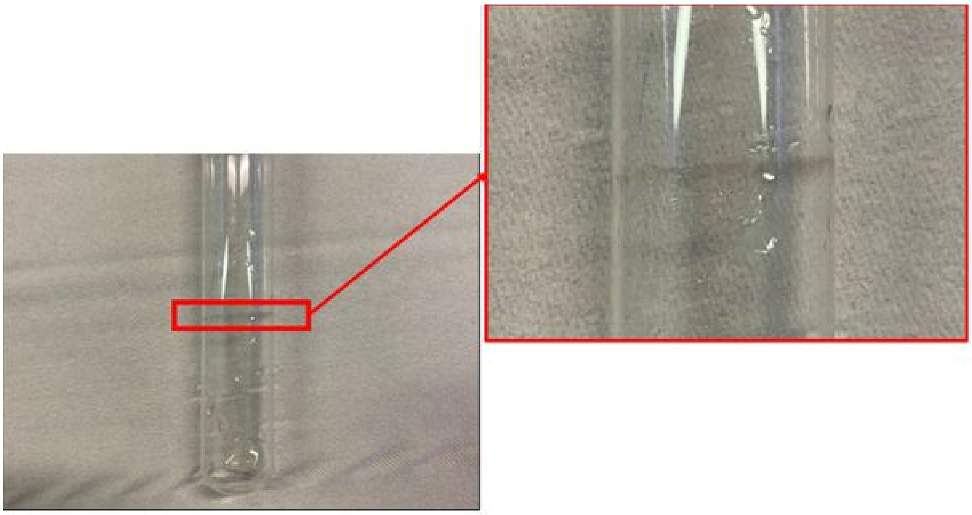

Fig. S3 Aggregation of AgNPs

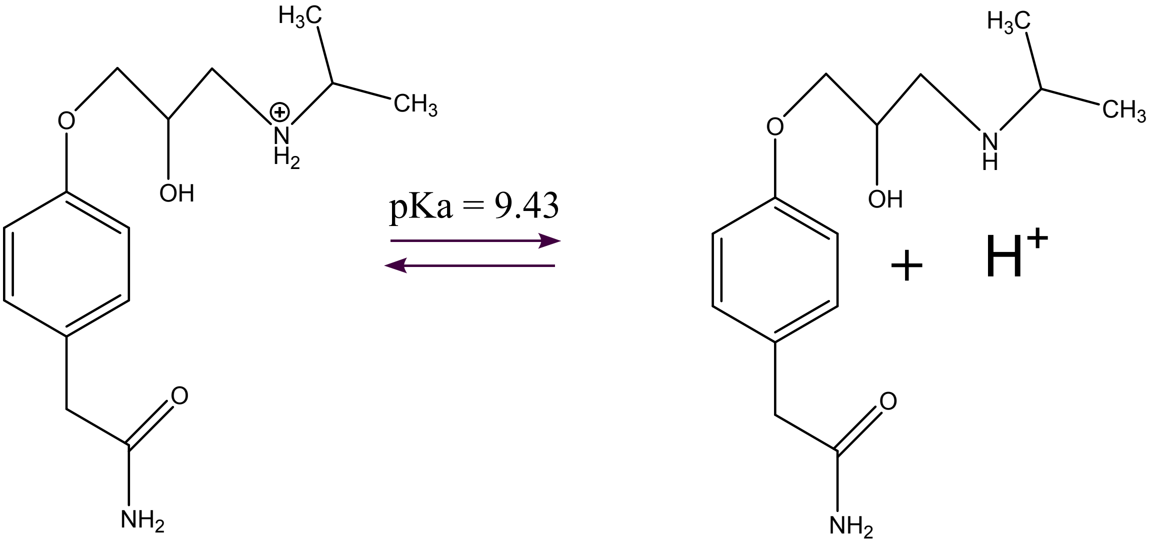

Fig. S4 ATN protonation equilibrium

Table S1. ANOVA test results for AuNPs removal

Fibers	Mean Value	p Value	F-value	F-critical
GLU2	5.05	≤ 0.05	21.26	4.06
GLU2-BET	1.07			
GLU2-LYS	9.41			
GLU-CYS	0.72			

Table S2. ANOVA test results for Atenolol removal

Fibers	Mean Value (%)	p Value	F-value	F-critical
GLU2	81.73	≤ 0.05	179.94	4.06
GLU2-BET	68.67			
GLU2-LYS	41.91			
GLU-CYS	63.94			

Table S3. ANOVA test results for CV removal

Fibers	Mean Value (%)	p Value	F-value	F-critical
GLU2	46.59	0.35	1.23	5.14
GLU2-BET	47.88			
GLU2-LYS	46.59			
GLU2-BET	47.88	≤ 0.05	2950.17	7.71
GLU-CYS	97.22			
